# Effects of a Transverse Field in Two Mixed-Spin Ising Bilayer Films

**DOI:** 10.3390/nano7090256

**Published:** 2017-09-04

**Authors:** Takahito Kaneyoshi

**Affiliations:** Graduate School of Information and Science, Nagoya University, 1-510, Kurosawadai, Midoriku, Nagoya 458-0003, Japan; kaneyosi@is.nagoya-u.ac.jp; Tel.: +81-528-766-607

**Keywords:** phase diagrams, magnetizations, compensation point, reentrant phenomena, mixed-spin Ising bilayer films

## Abstract

The magnetic properties (phase diagrams and magnetizations) of two mixed-spin Ising bilayer films with a transverse field are investigated by the use of the effective field theory with correlations. The systems consist of two magnetic atoms where spin-1/2 atoms are directed to the *z*-direction and only spin-1 atoms are canted from the *z*-direction by applying a transverse field. We examined how magnetization sign reversal can be realized in the system, due to the effects of the transverse field on the spin-1 atoms. The compensation point phenomena are found in both systems, depending on the selections of physical parameters. However, the reentrant phenomena are found only for one of the two systems.

## 1. Introduction

The theoretical research of various mixed-spin Ising (or transverse Ising) systems on honeycomb lattice has a long history and it is still a subject of active research. They have been examined by using various theoretical frameworks of mean-field theory (MFT), effective-field theory (EFT), the Green function method and the Monte Carlo simulation (MC). The studies of these systems are related to the experimental researches of molecular-based magnetic materials and nano-graphene films. In particular, a kind of molecular-based ferrimagnets, namely AFe^II^Fe^III^(C_2_O_4_)_3_ (A = N(*n* − C*_n_*H_2*n*+1_)_4_, *n* = 3–5), have been the subject under extensive theoretical discussions of mixed-spin Ising (or transverse Ising) systems, where spins of Fe^II^ and Fe^III^ atoms are respectively taken as 2 and 5/2 and they are coupled by a negative exchange interaction in each layer and two positive interactions between layers (see the references in the recent theoretical works [1,2,3,4]). For nano-graphene films, on the other hand, the magnetic properties of the ferromagnetic and antiferromagnetic two (or three) layers of honeycomb lattices described by the spin-1/2 Ising (or transverse Ising) models have been recently investigated by the use of the EFT [5,6,7]. The EFT [8,9] corresponds to the Zernike approximation [10] and it is believed to give more exact results than those of the MFT, since it automatically includes some correlations between a certain spin and the near-neighbor spins. Furthermore, the results obtained from the EFT have also the same topology as those obtained from the MC. In fact, the magnetic properties (magnetization, internal energy and so on) of a system are the same for the EFT and the MC, while the results obtained from the MC are smaller than those obtained from the EFT (for instance, see the works [11,12,13]). 

The aim of this work is to present another aspect of theoretical explanation for the appearance of negative magnetization in some molecular-based ferrimagnetic materials [14,15] on the basis of two layers of honeycomb lattices described by the mixed-spin (spin-1/2 and spin-1) Ising model, where the spin direction of spin-1/2 atoms is directed to the *z*-(or easy) direction and the spin direction of spin-1 atoms is canted from the *z*-direction, due to the presence of a transverse field. In [14], for instance, A in the above-described molecular-based ferrimagnets has been replaced by A = (XR_4_) with X = N, P and R = *n*-propyl, *n*-butyl, phenyl, instead of A = N(*n* − C*_n_*H_2*n*+1_)_4_, (*n* = 3–5). In these systems, only the system with A = NBu^n^_4_ has exhibited a negative magnetization at low-temperature. In this work, the two model systems are examined by the use of the EFT [16], in order to clarify whether such a negative magnetization can be realized within the present two model systems. In [16], the magnetizations of a mixed-spin system consisting of spin-1/2 and spin-1 atoms with different transverse fields have been examined. The temperature dependences of total magnetization in the system have shown typical ferrimagnetic behaviors usually observed in crystalline ferrimagnetic alloys A_p_B_1-p_, due to a strong negative A-B exchange interaction, where A and B are different magnetic atoms with different spins. The physical reason for this comes from the different spin canting of spin-1/2 and spin-1 atoms. 

In Section 2, the two systems are proposed. At first sight, they are very similar. Even within the theoretical formulations of the EFT, the basic equations obtaining the magnetic properties (phase diagram and magnetizations) are rather different between the two systems (system A and system B). In Section 3, the magnetic properties of system A are discussed by solving the formulations numerically. In Section 4, the magnetic properties of system B are obtained numerically. The last section is devoted to the conclusion.

## 2. Models and Formulation

**Figure 1:**
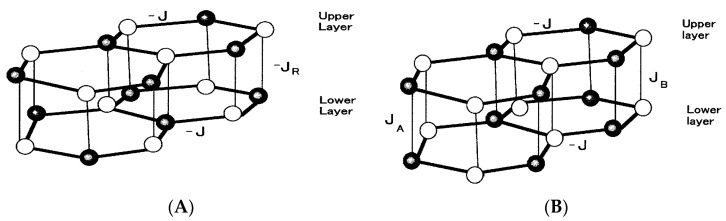
Schematic representations of two mixed spin Ising ferrimagnetic models on two (upper and lower) layered honeycomb lattices with spin-1/2 (white circle) and spin-1 (black circle) atoms. (**A**) Each Ising spin on upper layer is coupled to the corresponding same spin on the lower layer with an exchange interaction-*J_R_* (*J_R_* > 0.0). (**B**) Two exchange interactions (*J_A_* > 0.0 and *J_B_* > 0.0) exist between the two layers, depending on where the pair is located. *J_A_* is the interlayer coupling between the spin-1 atoms and *J_B_* is the interlayer coupling between the spin-1/2 atoms.

We consider the two systems consisting of two layers, as depicted in Figure 1. The structure of each system is the honeycomb lattice. The white and black circles on each figure represent respectively the magnetic atoms with spin-1/2 and the magnetic atoms with spin-1 and transverse field Ω. The spins (white and black circles) on each layer are coupled by a nearest-neighbor exchange interaction-*J* (*J* > 0.0). In Figure 1A, each Ising spin on the upper layer is coupled to the corresponding same spin on the lower layer with an exchange interaction-*J_R_* (*J_R_* > 0.0). In Figure 1B, on the other hand, two exchange interactions (*J_A_* > 0.0 and *J_B_* > 0.0) exist between the two layers, depending on where the pair is located. *J_A_* is the interlayer coupling between the spin-1 atoms and *J_B_* is the interlayer coupling between the spin-1/2 atoms. The Hamiltonian of Figure 1A (or system A) is given by

(1a) $$H\;=\;J\;\underset{\left(ij\right)}{\Sigma }\;\sigma_{i}{}^{Z}S_{j}{}^{Z}+\;J_{R}\underset{\left(ij\right)}{\Sigma }\;\sigma_{i}{}^{Z}S_{j}{}^{Z}-\;\Omega \;\underset{j}{\Sigma }\;S_{j}{}^{X}$$

where *σ_i_^Z^* represents the spin-1/2 operator with *σ_i_^Z^* = ±1/2 and *S_j_^α^* (*α* = *Z* and *X*) is the spin-1 operator with *S_j_^Z^* = ±1 and 0. The first and the second terms in (1a) represent the contributions from the intra-layer and inter-layer interactions. The last term shows the effect of a transverse field at each spin-1 atom. On the other hand, the Hamiltonian of Figure 1B (or system B) can be represented by

(1b) $$H\;=\;J\;\underset{\left(ij\right)}{\Sigma }\;\sigma_{i}{}^{Z}S_{j}{}^{Z}-\;J_{A}\underset{\left(kl\right)}{\Sigma }\;\sigma_{k}{}^{Z}S_{l}{}^{Z}-\;J_{B}\underset{\left(mn\right)}{\Sigma }\;\sigma_{m}{}^{Z}S_{n}{}^{Z}-\;\Omega \;\underset{{}^{j}}{\Sigma }\;S_{j}{}^{X}$$

where the second and third terms show the contributions from the inter-layer interactions. 

The total longitudinal magnetization (*m_T_* = *m_T_^Z^*) per site in each system (A or B) is defined as

*m_T_* = [*m_A_* + *m_B_*]/2.0 (2)

where *m_A_* = *m_A_^Z^* = <*S_j_^Z^*> and *m_B_* = *m_B_^Z^* = <*σ_i_^Z^*>. Within the EFT [8,9,16], the *m_A_* and *m_B_*in the system A (Figure 1A) are given by

*m_A_* = [*cosh*(*C*/2.0) − 2.0 *m_B_sinh*(*C*/2.0)]^3^ [*cosh*(*R*/2.0) − 2.0 *m_B_ sinh*(*R*/2.0)] *F*(*x*)|_*x*=0_ (3)

*q_A_* = [*cosh*(*C*/2.0) − 2.0 *m_B_sinh*(*C*/2.0)]^3^ [*cosh*(*R*/2.0) − 2.0 *m_B_ sinh*(*R*/2.0)] *G*(*x*)|_*x*=−0_ (4)

and

*m_B_* = [*q_A_* {*cosh*(*C*) − 1.0} + 1.0 − *m_A_ sinh*(*C*)]^3^ [*q_A_* {*cosh*(*R*) − 1.0} + 1.0 − *m_A_ sinh*(*R*)] *f*(*x*)|_*x*=0_ (5)

where *q_A_* = < (*S_j_^Z^*)^2^>, *C* = *J·D* and *R* = *J_R_·D*. *D* = *∂*/*∂x* is the differential operator. Here, the functions *F*(*x*)*, G*(*x*) and *f*(*x*) are defined by

*F*(*x*) = 2.0 *x sinh*(*e*(*x*)*β*)/[*e*(*x*) {1.0 + 2.0 *cosh*(*βe*(*x*))}] (6)

*G* (*x*) = [*Ω*^2^ + {*Ω*^2^ + 2.0 *x*^2^} *cosh*(*βe*(*x*))]/[*e*(*x*)^2^ {1.0 + 2.0 *cosh*(*βe*(*x*))}] (7)

*f*(*x*) = *tanh*(*βx*/2.0)/2.0 (8)

with

*e*(*x*) = [*Ω*^2^ + *x*^2^]^1/2^ (9)

where *β* = 1.0/*k_B_T*. 

For the system B in Figure 1B, the *m_A_* and *m_B_* are given by

*m_A_* = [*cosh*(*C*/2.0) − 2.0 *m_B_sinh*(*C*/2.0)]^3^ [*q_A_* {*cosh*(*A*) − 1.0} + 1.0 + *m_A_ sinh*(*A*)] *F*(*x*)|_*x*=0_ *q_A_* = [*cosh*(*C*/2.0) − 2.0* m_B_ sinh*(*C*/2.0)]^3^ [*q_A_* {*cosh*(*A*) − 1.0} + 1.0 + *m_A_sinh*(*A*)] *G*(*x*)|_*x*=−0_ (10)

and

*m_B_* = [*q_A_* {*cosh* (*C*) − 1.0} + 1.0 − *m_A_sinh*(*C*)]^3^ [*cosh*(*B*/2.0) + 2.0 *m_B_sinh*(*B*/2.0)] *f*(*x*)|_*x*=0_ (11)

where *A* = *J_A_·D* and *B* = *J_B_·D*. 

The phase diagrams (or transition temperature) in the two systems can be determined by expanding the coupled equations of *m_A_* and *m_B_* in each system (A or B) linearly. The transition temperature of system A can be obtained from the relation

[3.0 *K*_1_ + *K*_2_] [6.0 *K*_3_ + 2.0 *K*_4_] − 1.0 = 0.0 (12)

where the coefficients *K_n_* (*n* = 1–4) are given by

*K*_1_ = *sinh*(*C*) [*q_A_* {*cosh*(*C*) − 1.0} + 1.0]^2^ [*q_A_* {*sinh*(*R*) − 1.0} + 1.0] *f*(*x*)|_*x*=0_ *K*_2_ = *sinh*(*R*) [*q_A_* {*cosh*(*C*) − 1.0} + 1.0]^3^*f*(*x*)|_*x*=0_ *K*_3_ = *cosh*^2^(*C*/2.0) *sinh*(*C*/2.0) *cosh*(*R*/2.0) *F*(*x*)|_*x*=0_ *K*_4_ = *cosh*^3^(*C*/2.0) *sinh*(*R*/2.0) *F*(*x*)|_*x*=0_ (13)

with

*q_A_* = *cosh*^3^(*C*/2.0) *cosh*(*R*/2.0) *G*(*x*)|_*x*=0_.

The transition temperature of system B can be obtained from the relation

[*L*_1_ − 1.0] [2.0 L_2_ − 1.0] − 18.0 *L*_3_*L*_4_ = 0.0 (14)

The coefficients Ln (*n* = 1–4) in (14) are given by

*L*_1_ = *cosh*^3^(*C*/2.0) *sinh*(*A*) *F*(*x*)|_*x*=0_ *L*_2_ = *sinh*(*B*/2.0) [*q_A_* {*cosh*(*C*) − 1.0} + 1.0]^3^*f*(*x*)*|_x_*_=0_ *L*_3_ = *cosh*(*C*/2.0) *sinh*(*C*/2.0) [*q_A_* {*cosh*(*A*) − 1.0} + 1.0] *F*(*x*)|_*x*=0_ *L*_4_ = *sinh*(*C*) *cosh*(*B*/2.0) [*q_A_* {*cosh*(*C*) − 1.0} + 1.0]^2^*f*(*x*)|_*x*=0_ (15)

with

*q_A_* = *D*_1_/(1.0 + *D*_1_ − *D*_2_) (16)

where *D*_1_ and *D*_2_ are defined as

*D*_1_ = *cosh*^3^(*C*/2.0) *G*(*x*)|_*x*=0_ *D*_2_ = *cosh*^3^(*C*/2.0) *cosh*(*A*) *G*(*x*)|_*x*=0_ (17)

At this point, it is important to note that only spin-1 magnetic atoms in the two systems are affected by a transverse field *Ω* and hence they are canted from the *z*-direction, when *Ω* takes a finite value. However, spin-1/2 atoms in them are always directed to the *z*-direction. At first sight, the two systems given in Figure 1 seem to be physically equivalent. As discussed above, however, the theoretical formulations of the two systems are rather different even within the theoretical formulation of the EFT. Under these conditions, it may be important to know what phenomena can be obtained in the two systems. Furthermore, it is also important to know whether the phenomena are similar between the two systems or some differences may be found in the phenomena of the two systems. As far as we know, these problems have not been discussed. In the following, the magnetic properties (phase diagram and thermal variation of magnetizations) in system A (or Figure 1A) are examined in Section 3. In Section 4, such magnetic properties of system B (or Figure 1B) are discussed. 

## 3. The Magnetic Properties of System A

At first, let us define the parameters, *t*, *h* and *r* as

*t* = *k_B_T/J*, *h* = *Ω/J* and *r* = *J_R_/J* (18)

**Figure 2:**
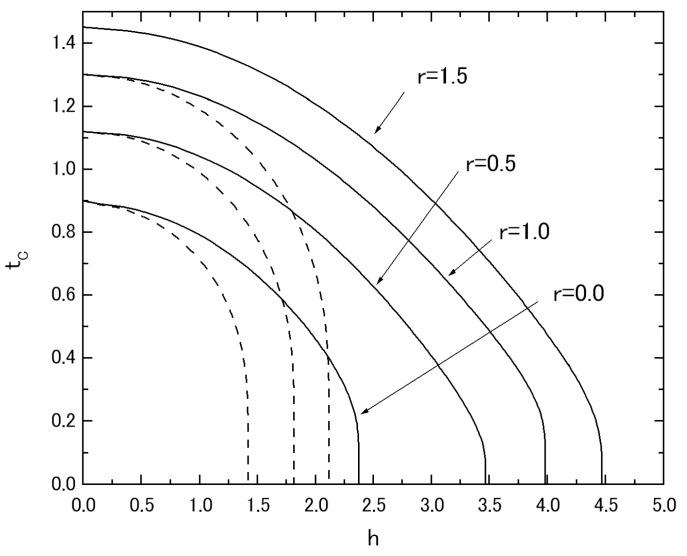
The phase diagram (*t_C_* versus *h* plot) for the system A, when the value of *r* is changed from *r* = 0.0 to *r* = 1.5.

In Figure 2, the phase diagram (*T_C_* versus *h* plot) in the system A is given by changing the value of *r* from *r* = 0, 0 to *r* = 1.5. In the figure, the dashed lines represent the results of the *t_C_* (*t_C_* = *k_B_T_C_*/*J*) versus *h* plot, when the system A is described by the following Hamiltonian,

$$H\;=\;J\;\underset{\left(ij\right)}{\Sigma }\;\sigma_{i}{}^{Z}S_{j}{}^{Z}+\;J_{R}\underset{\left(ij\right)}{\Sigma }\;\sigma_{i}{}^{Z}S_{j}{}^{Z}-\;\Omega \;\left(\underset{I}{\Sigma }\sigma_{i}{}^{X}+\;\underset{j}{\Sigma }\;S_{j}{}^{X}\right)$$

namely when both spin-1/2 and spin-1 atoms have the same transverse field *Ω*, instead of the present system with the Hamiltonian (1,a). At this stage, one should notice that the different behavior between the solid curve and the dashed curve in the system with the same value of *r* makes an important contribution to the appearance of negative magnetization at a low temperature. 

**Figure 3:**
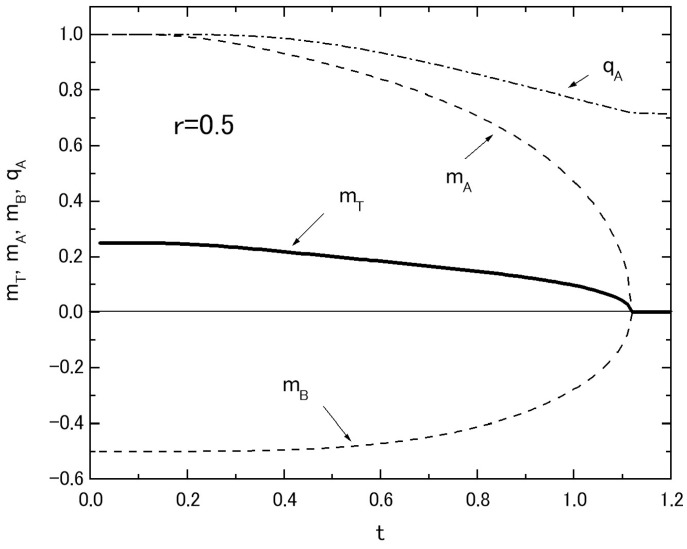
The temperature dependences of *m_T_* (solid curve), *m_A_* (dashed curve), *m_B_* (dotted curve) and *q_A_* (dot-dashed curve) in the system A with a fixed value of *r* = 0.5, when the value of *h* is given by *h* = 0.0.

In Figure 3, the temperature dependences of magnetizations in the system with fixed values of *r* = 0.5 and *h* = 0.0 are plotted, where *m_T_*, *m_A_* and *m_B_* are respectively represented by solid, dashed and dotted curves. The thermal variation of *q_A_* is also plotted in Figure 3 by the dot-dashed curve. As is seen from the figure, *m_A_* and *m_B_* take respectively a positive and negative value in the whole region of *T* below its *T_C_*. Here, one should notice that the value of *r* = 0.5 is particularly selected, since the distance between the two layers are supposed to be larger than the lattice interval in each layer [14,15] and hence the exchange interaction *J _R_* is assumed to be weaker than that of intra-layer interaction *J*. 

**Figure 4:**
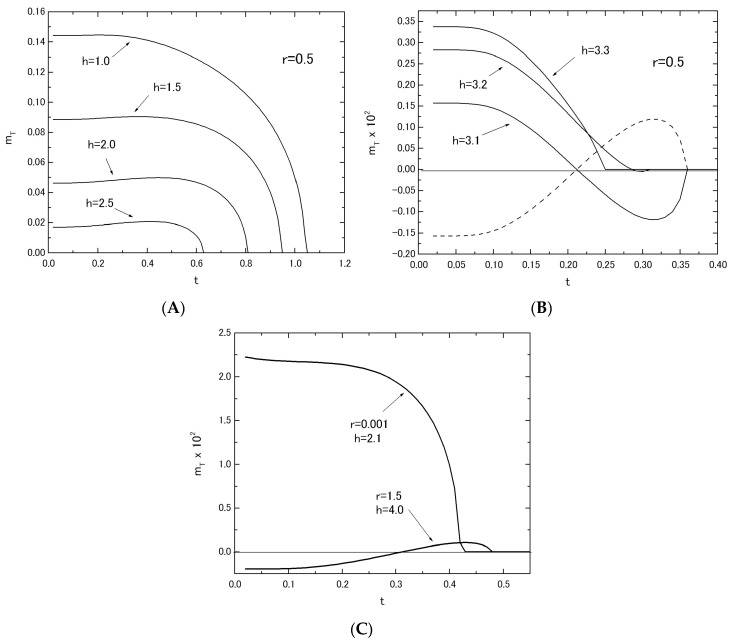
The temperature dependences of *m_T_* in the system A; the value of *r* is fixed at *r* = 0.5 (**A**,**B**). The value of *h* is changed from *h* = 1.0 to *h* = 2.5 in (**A**) and from *h* = 3.1 to *h* = 3.3 in (**B**). The dashed curve represents the result of the system with *r* = 0.5 and *h* = 3.1, when the signs of *m_A_* and *m_B_* are changed from the case of (*m_A_* > 0.0, *m_B_* < 0.0) to the case of (*m_A_* < 0.0, *m_B_* > 0.0). The thermal variations of *m_T_* are given, when the two set values (*r* and *h*) are selected as (*r* = 0.001 and *h* = 2.1) and (*r* = 1.5 and *h* = 4.0) (**C**).

In Figure 4A,B, the thermal variations of *m_T_* in the system with *r* = 0.5 are shown by changing the value of *h* from *h* = 1.0 to *h* = 3.3. As shown in Figure 4A, the behavior of *m_T_* may change from the Q-type to the P-type with the increase of h. In Figure 4B, the curves labeled *h* = 3.1 and *h* = 3.2 exhibit a compensation point below their transition temperatures (or the N-type), although the curve labeled *h* = 3.3 takes the Q-type behavior. The nomenclature of Q-, P- and N-types in ferrimagnetism has been used (see [17]). In Figure 4B, the dashed curve represents the thermal variation of *m_T_* in the system with *r* = 0.5, when the signs of *m_A_* and *m_B_* are changed from the case of (*m_A_* > 0.0, *m_B_* < 0.0) to the case of (*m_A_* < 0.0, *m_B_* > 0.0). In Figure 4C, on the other hand, it is shown that the same behavior of m_T_ as the dashed curve in Figure 4B can be easily obtained by selecting the reasonable parameters of r and h (such as the curve labeled *r* = 1.5 and *h* = 4.0), even when *m_A_* > 0.0 and *m_B_* < 0.0. The curve clearly exhibits a negative magnetization in the low temperature region. The curve labeled (*r* = 0.001 and *h* = 2.1) in Figure 4C exhibits the Q-type behavior. The results shown in Figure 4B,C are similar to those obtained in [14,15].

## 4. The Magnetic Properties of System B

From the structural aspect of the present two systems, system B seems to be more realistic than system A [14,15], although the spin structure is more complicated than that of system A. In fact, for the inter-layer interaction, there exist two exchange interactions *J_A_* and *J_B_* in system B, while there exists only one exchange interaction *J_R_* in system A. Accordingly, let us define the following parameters *t*, *h*, *r* and *s* as

*t* = *k_B_T*/*J*, *h* = *Ω*/*J*, *r* = *J_A_*/*J* and *s* = *J_B_*/*J* (19)

**Figure 5:**
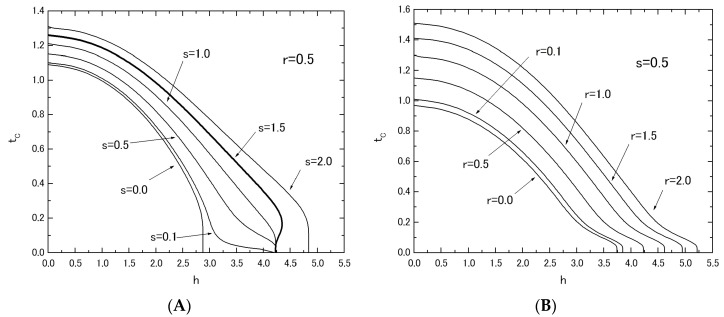
The phase diagrams (*t_C_* versus *h* plot) for the system B; The value of *r* is fixed at *r* = 0.5 and the value of *s* is changed from *s* = 0.0 to *s* = 2.0 (**A**). The value of *s* is fixed at *s* = 0.5 and the value of *r* is changed from *r* = 0.0 to *r* = 2.0 (**B**).

**Figure 6:**
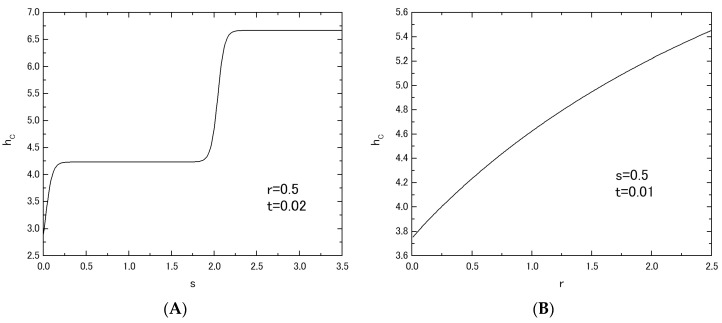
The variations of *h_C_* in the system B; the variation of *h_C_* in the system with *r* = 0.5 and *t* = 0.02 is given, when the value of *s* is changed from *s* = 0.0 to *s* = 3.5 (**A**). The variation of *h_C_* in the system with *s* = 0.5 and *t* = 0.01 is given, when the value of *r* is changed from *r* = 0.0 to *r* = 2.5 (**B**).

**Figure 7:**
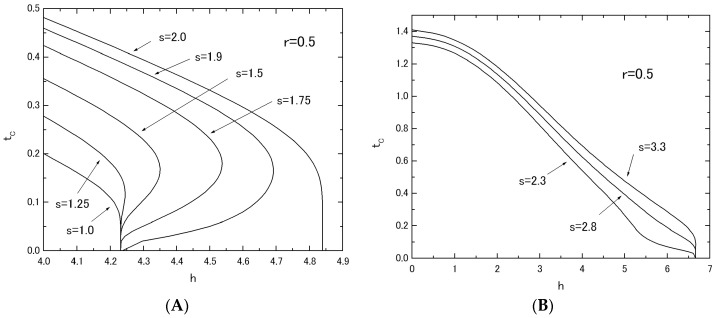
The phase diagrams (*t_C_* versus *h* plot) for the system B with *r* = 0.5; the value of *s* is changed from *s* = 1.0 to *s* = 2.0 (**A**). Three values of *s* are selected as *s* = 2.3, *s* = 2.8 and *s* = 3.3 (**B**).

Figure 5 shows the phase diagrams (*t_C_* versus *h* plot) of the system B, when the value of *r* (or *s*) is fixed at *r* = 0.5 in Figure 5A (or *s* = 0.5 in Figure 5B) and the value of *s* (or *r*) is changed from *s* = 0.0 to *s* = 2.0 (or from *r* = 0.0 to *r* = 2.0). As is seen from these figures, the behavior of *t_C_* curves in Figure 5A is completely different from that in Figure 5B as well as the results of Figure 2 for system A. In Figure 5A, the reentrant phenomenon has been obtained for the curve labeled *r* = 1.5 (or bold solid curve). However, the same phenomenon has not been obtained in Figure 5B. In order to clarify these differences between Figure 5A and Figure 5B, the *h_C_* versus *s* (or *r*) plot has been examined in Figure 6. The value of *h_C_* can be obtained from the results of Figure 5 as a critical value of *h* at which the *t_C_* curve reduces to zero. Figure 6A shows the *h_C_* versus s plot, when the value of *r* is fixed at *r* = 0.5. Figure 6B shows the *h_C_* versus *r* plot, when the value of *s* is fixed at *s* = 0.5. One can easily find clear differences between Figure 6A and Figure 6B. As shown in Figure 7A, only the flat region of *h_C_* in Figure 6A between *h_C_* = 1.0 and *h_C_* = 2.0 may exhibit the reentrant phenomenon, although, for the flat region of *h_C_* > 2.0 of Figure 6A, the phenomenon has not been found, as shown in Figure 7B. In Figure 6B, on the other hand, the value of *h_C_* increases simply with the increase of *r*. A phenomenon similar to that of Figure 7B can be also obtained for system A, when the value of *h_C_* is plotted as a function of *r* by using the results of Figure 2. 

**Figure 8:**
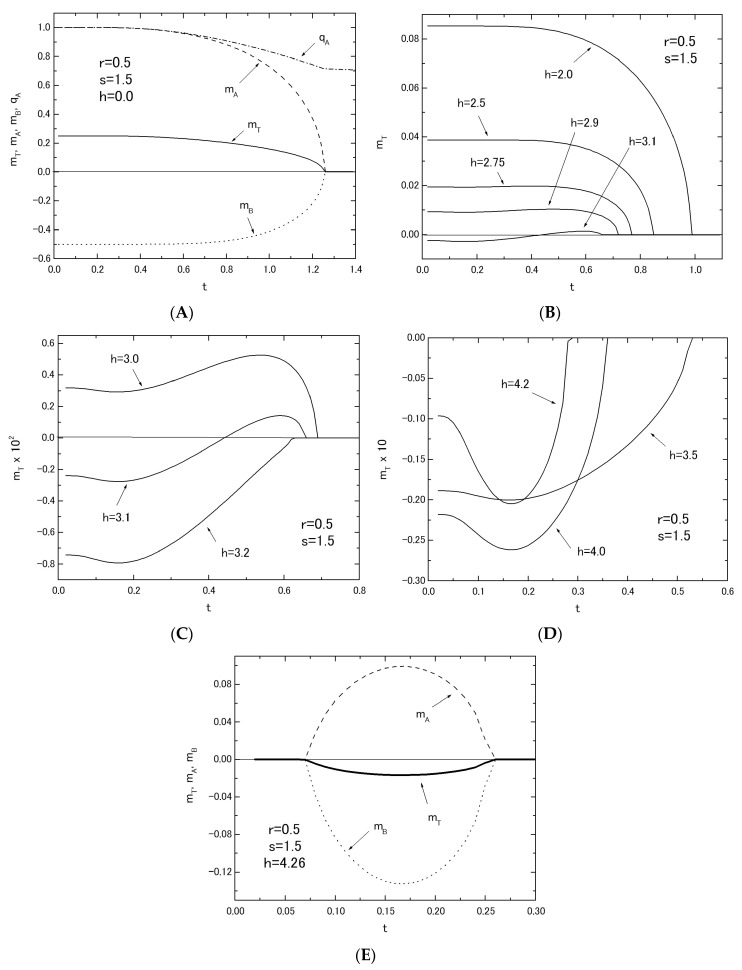
The temperature dependences of *m_T_* in the system B with *r* = 0.5 and *s* = 1.5; the thermal variations of *m_T_* (solid curve), *m_A_* (dashed curve), *m_B_* (dotted curve) and *q_A_* (dot-dashed curve) are depicted, when the value of *h* is given by *h* = 0.0 (**A**). The thermal variations of *m_T_* are given, changing the value of *h* from *h* = 2.0 to *h* = 3.1 (**B**). The thermal variations of *m_T_* are given by selecting the three values of *h* (**C**,**D**). The reentrant phenomena of magnetizations are presented by selecting the value of *h* as *h* = 4.26 (**E**).

In Figure 8, the thermal variations of *m_T_* in the system with fixed values of *r* = 0.5 and *s* = 1.5 are given by changing the value of *h* from *h* = 0.0 to *h* = 4.26, in order to clarify whether the above predictions of phase diagrams are correct. In Figure 5A, the reentrant phenomenon has been obtained in the region of 4.22 < *h* < 4.36 for the system with *r* = 0.5 and *s* = 1.5. In Figure 8E, the thermal variations of *m_T_* (solid curve), *m_A_* (dashed curve) and *m_B_* (dotted curve) are plotted by selecting the value of *h* = 4.26 from the region of 4.22 < *h* < 4.36. They clearly exhibit the reentrant phenomenon, as predicted in the phase diagrams. At this place, one should notice that the reentrant phenomenon is not a peculiar behavior and it has often been found for some finite-size magnetic systems described by some transverse Ising models, as discussed in some recent works [18,19,20,21]. As is seen from Figure 8A to Figure 8D, the thermal variations of *m_T_* in the system may change a positive magnetization to a negative magnetization with the increase of h and may exhibit some characteristic ferrimagnetic behaviors, such as Q-, P-, N- and M-types, although some of them could not be classified by the nomenclature of ferrimagnetism [17]. In fact, the thermal variations of *m_T_* for the curves labeled *h* = 3.0 and *h* = 3.1 in Figure 8C are novel types. They start to decrease from the saturation magnetization at *t* = 0.0, exhibit a broad minimum, a broad maximum with the increase of *t* and then reduce to zero at their transition temperatures. 

**Figure 9:**
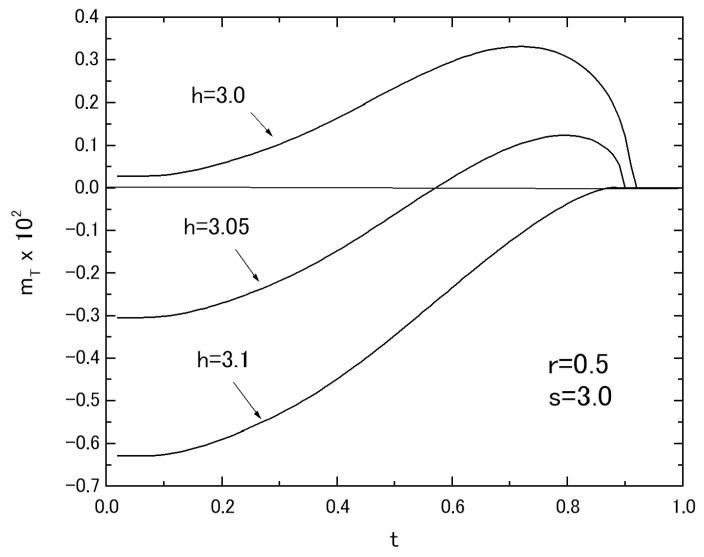
The temperature dependences of *m_T_* in the system B with *r* = 0.5 and *s* = 3.0, when the three values of *h* are selected.

**Figure 10:**
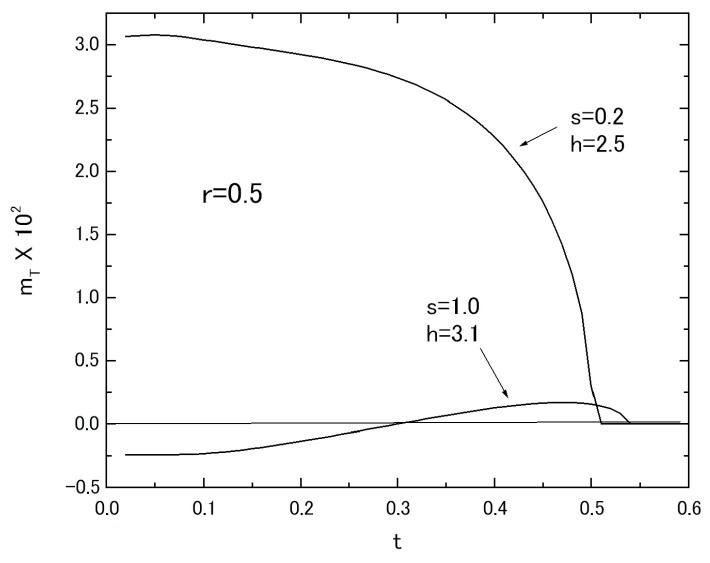
The thermal variations of *m_T_* in the system with *r* = 0.5 are given, when the two set values (*s* and *h*) are selected as (*s* = 0.2 and *h* = 2.5) and (*s* = 1.0 and *h* = 3.1).

In relation to the results of Figure 7B, the thermal variations of *m_T_* in the system with fixed value of *r* = 0.5 and *s* = 3.0 are plotted in Figure 9, selecting the three values of *h*. The curve labeled *h* = 3.0 shows the L-type behavior. The curve labeled *h* = 3.1 exhibits the N-type behavior. The curve labeled *h* = 3.2 represents the M-type behavior. The thermal variations of *m_T_*, corresponding to the results of *m_T_* depicted in Figure 4C for the system A with *r* = 0.5, are depicted in Figure 10, selecting the two sets of *s* and *h* in the system B with *r* = 0.5. The results shown in Figure 9 and Figure 10 are also similar to those obtained in [14,15].

**Figure 11:**
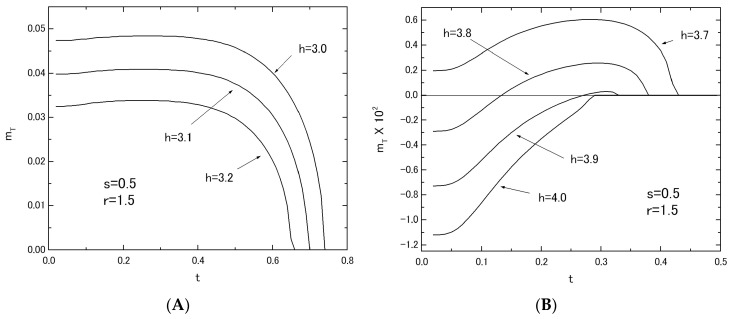
The temperature dependences of *m_T_* in the system B with *s* = 0.5 and *r* = 1.5; the value of *h* is changed from *h* = 3.0 to *h* = 3.2 (**A**) and from *h* = 3.7 to *h* = 4.0 (**B**).

Finally, the thermal variations of *m_T_* in the system with fixed values of *s* = 0.5 and *r* = 1.5 are given in Figure 11, changing the value of *h* from *h* = 3.0 to *h* = 4.0. These results can be compared with those of Figure 8B,C for the system with *r* = 0.5 and *s* = 1.5. 

## 5. Conclusions

In this work, within the theoretical framework of the effective-field theory with correlations (EFT), we have investigated the phase diagrams and the magnetizations in the two mixed spin Ising systems consisting of two layers with honeycomb lattice. As shown in Figure 1, at first sight, their magnetic properties seem to be similar to each other, since they have the same intra-layer spin structure. As shown in Figure 5A, Figure 6A, Figure 7A and Figure 8E, the big differences between the two systems can be seen in the appearance of the reentrant phenomenon and the characteristic behavior of *h_C_* in system B. In particular, one should also notice that such characteristic features have been obtained in system B, only when the value of *J_A_* is fixed and the value of *J_B_* is changed. In fact, as shown in Figure 5B, such characteristic phenomena have not been obtained, when the value of *J_B_* is fixed and the value of *J_A_* is changed. 

From the numerical examinations of *m_T_* given in Section 3 and Section 4, all types of behaviors normally obtained for the thermal variation of *m_T_* in bulk ferrimagntic materials, such as the Q-, P-, N-, L- and M-types, can be found in the present two systems. As shown in Figure 8C, furthermore, novel types have been obtained for the thermal variation of *m_T_* in system B.

In the present work, we have examined the magnetic properties of the two systems consisting of spin-1/2 and spin-1 atoms. Realistically, such systems should be constructed from spin-2 and spin-5/2 atoms, in order to compare with experimental data in molecular-based ferriagnetic materials [14,15]. The numerical results obtained from such systems are probably similar to the present ones obtained in Section 3 and Section 4.
